# Dentoalveolar changes observed after the use of customized bonded Shark-Tooth-Like Spurs (JAWs) in adult patients with anterior open bite

**DOI:** 10.1590/2177-6709.27.5.e2220448.oar

**Published:** 2023-01-06

**Authors:** Rungrawee KRAISIRIDEJ, Boonsiva SUZUKI, Eduardo Yugo SUZUKI

**Affiliations:** 1 Bangkokthonburi University, Faculty of Dentistry, Department of Orthodontics (Bangkok, Thailand).

**Keywords:** Adult, Orthodontics, Open bite, Spurs, Thrusting

## Abstract

**Introduction:**

Tongue spurs have been successfully used for the early treatment of anterior open bite (AOB). However, according to our knowledge, their effectiveness in the treatment of adults has not been evaluated.

**Objectives:**

The purpose of the study was to assess the dentoalveolar changes observed after the use of customized bonded shark-tooth-like spurs (JAWs) in adults with AOB.

**Methods:**

Twenty-three adults (22.1±4.4 years) with AOB were selected for the treatment. JAWs made from compomer cement were bonded on the lingual surfaces of the maxillary and mandibular anterior teeth to correct tongue-thrusting. Lateral cephalograms and 3D digital models were obtained to evaluate dentoalveolar features observed before and at three time points after JAWs use. Paired *t*-test and repeated measure ANOVA tests were used to compare dentoalveolar changes, and Pearson’s correlation was used to analyze the association of dentoalveolar changes and overbite changes. The significance level was set at *p*<0.05.

**Results:**

Significant 3D dentoalveolar changes were observed after the three months of treatment with JAWs. Improvement of overbite (1.0±0.6 mm) and overjet (0.2±0.3 mm), combined with a retroclination of maxillary (3.0±3.0°) and mandibular (2.2±2.7°) incisors, were observed (*p*<0.05). Moreover, a significant decrease in anterior dental arch width in both maxillary and mandibular arches (0.4±0.4 mm; 0.3±0.3 mm, respectively), and an increase of posterior maxillary (0.1±0.2 mm) dental arch width were observed (*p*<0.05). These significant changes occurred in the first month after the JAWs use. A significant correlation was found between the initial arch length discrepancy and the amount of overbite correction (r=0.456, *p*<0.05).

**Conclusions:**

Dentoalveolar changes occurred after the first-month therapy with JAWs. The retroclination of the anterior teeth combined with the expansion of posterior teeth suggests a posterosuperior change in the tongue position. These changes were beneficial for the treatment of AOB in adult patients.

## RESUMO

**Introdução:**

Esporões linguais têm sido utilizados com sucesso para o tratamento precoce da mordida aberta anterior (MAA). No entanto, de acordo com nosso conhecimento, sua efetividade no tratamento em adultos ainda não foi avaliada.

**Objetivo:**

O objetivo do presente estudo foi avaliar as mudanças dentoalveolares observadas após o uso de esporões colados customizados JAWs (tipo dente de tubarão) em adultos com MAA.

**Métodos:**

Vinte e três adultos (idade média de 22,1±4,4 anos) com MAA foram selecionados para tratamento. Esporões confeccionados de compômero foram colados na superfície lingual dos dentes anteriores superiores e inferiores, para corrigir a interposição lingual. Radiografias laterais e modelos digitais 3D foram obtidos para avaliar as características dentoalveolares observadas antes do uso dos esporões e em três tempos distintos após seu uso. O teste*t* pareado e testes ANOVA para medições repetidas foram utilizados para comparar as mudanças dentoalveolares, e a correlação de Pearson foi utilizada para analisar a associação entre as mudanças dentoalveolares e as mudanças na sobremordida. O nível de significância foi estabelecido em *p*<0,05.

**Resultados:**

Foram observadas mudanças dentoalveolares 3D significativas após três meses de tratamento com os esporões. Foram observadas melhorias na sobremordida (1,0±0,6 mm) e sobressaliência (0,2±0,3 mm), associadas à retroinclinação dos incisivos superiores (3,0±3,0°) e inferiores (2,2±2,7°) (*p*<0,05). Além disso, foi observada diminuição significativa na largura anterior das arcadas superior e inferior (0,4±0,4 mm; 0,3±0,3 mm, respectivamente) e um aumento na largura posterior da arcada dentária superior (0,1±0,2 mm) (*p*<0,05). Essas mudanças significativas ocorreram no primeiro mês após o uso dos esporões. Foi encontrada uma correlação significativa entre a discrepância inicial no comprimento da arcada e a quantidade de correção da sobremordida (r=0,456, *p*<0,05).

**Conclusões:**

Mudanças dentoalveolares ocorreram após o primeiro mês de tratamento com os esporões. A retroinclinação dos dentes anteriores combinada com a expansão dos dentes posteriores sugere uma mudança posterossuperior na posição da língua. Essas mudanças foram benéficas para o tratamento da MAA em pacientes adultos.

## INTRODUCTION

Anterior open bite (AOB) is characterized by the open vertical dimension or the presence of negative overbite between the incisal edges of the maxillary and mandibular anterior teeth when the posterior teeth are in occlusion.^1^ ^,^ ^2^ AOB is one of the most challenging malocclusions to be treated due to the high tendency to relapse.^3^

The unbalanced neuromuscular functions related to the tongue are considered the main factor associated with AOB.^1^ ^,^ ^4^ ^,^ ^5^ Consequently, various treatment methods have been proposed to correct the tongue habits, such as the surgical reduction,^6^ the use of a tongue elevator,^7^ tongue crib,^5^ ^,^ ^8^ ^,^ ^9^ myofunctional therapy,^10^ ^,^ ^11^ and tongue spurs.^5^ ^,^ ^12^ ^-^ ^16^ However, the use of tongue spurs has shown various clinical advantages, since they are simple to insert and can be used in conjunction with fixed orthodontic appliances.^13^ ^,^ ^14^

Tongue spurs were initially described by Rogers^15^ for controlling and correcting tongue dysfunction or incorrect posture. Justus^13^ has reported that an immediate nociceptive or proprioceptive reflex is established to a new rest posture and swallowing mode after spur use. A continuous biofeedback mechanism, created to inform the patient about the incorrect tongue position, has been reported by Meyer-Marcotty et al.^17^ A neurophysiological adaptation process is set after using tongue spurs. In comparison to spurs, other treatment modalities, such as cribs, are passive restraints, whereas myofunctional treatment controls the false tongue position and function, particularly in conscious patients.

For the early treatment of AOB, several studies have described significant changes resulting from different AOB treatment modalities in children,^3^ ^,^ ^12^ ^,^ ^18^ ^,^ ^19^ due to their growth potential. Cassis et al.^12^ evaluated the effects of the spurs with a chin cup in treating patients with AOB in the mixed dentition. Significant dentoalveolar changes that included the increase in dentoalveolar development of the maxillary and mandibular incisors, with correction of the overbite, were observed. Additionally, in isolated spur studies, dentoalveolar changes have been reported.^3^ ^,^ ^17^ ^,^ ^20^ ^,^ ^21^ Canuto et al.^3^ studied patients with AOB in the mixed dentition during early treatment, and stated that there was increased vertical dentoalveolar development of the maxillary incisors after spur treatment, whether the bonded type or conventional type of spur was employed. Meyer-Marcotty et al.^20^ also reported increases in overbite after utilization of the fixed palatal spur appliance, with no significant influence on the inclination of the jaw bases.

Although the use of tongue spurs has been successfully applied for the correction of tongue habits in young patients, with reported dentoalveolar changes, the effectiveness of this therapy in adult patients has never been assessed, as far as we are concerned. Therefore, the present study aimed at evaluating the dentoalveolar effects observed after the use of a customized shark-tooth-like spur (JAWs) made of compomer cement, and bonded on the lingual surfaces of the maxillary and mandibular anterior teeth, to correct the tongue-thrusting habit, thus providing a new tongue position and function to AOB patients. The hypothesis tested was that the use of JAWs would be an alternative approach to correct the tongue-thrusting habit in adult patients with AOB.

Therefore, the purpose of this study was to evaluate the dentoalveolar changes observed after the use of JAWs in adult patients with AOB.

## MATERIAL AND METHODS

### SUBJECTS

Twenty-three patients (17 females and 6 males) were recruited at the Graduate Clinic, Department of Orthodontics, Faculty of Dentistry, Bangkokthonburi University (mean age 22.1±4.4 years), diagnosed with AOB with tongue-thrusting swallowing habit, from October 2016 to December 2018. The pretreatment mean overbite was -1.4 ± 1.4 mm and mean overjet was 2.2 ± 2.1 mm. The participants were treated with customized JAWs before orthodontic corrective treatment. 

The sample size calculation (n ≥ 20) was determined using G*Power v. 3.1.9.7 software (Franz Faul, University of Kiel, Kiel, Schleswig-Holstein, Germany), with the effect size = 0.97 derived from the preliminary data, as reported by Meyer-Marcotty et al.^17^, α = 0.05 and Power (1-β) = 0.95 to detect a mean difference of 1.95 mm in overbite change between the groups, with an estimated standard deviation of 1.47 mm.

The inclusion criteria were: 1) adult patients with at least 18 years of age at the time of enrollment; 2) AOB defined as the absence of vertical overlap between one or more incisors in the opposing arches; 3) an anterior resting tongue posture and a tongue-thrusting swallowing pattern. This study was approved by the Human Ethics Committee of Bangkokthonburi University (approval number: 6/2561).

### EVALUATION OF THE TONGUE DURING SWALLOWING

#### AND IN THE RESTING POSITION

**Figure 1:**
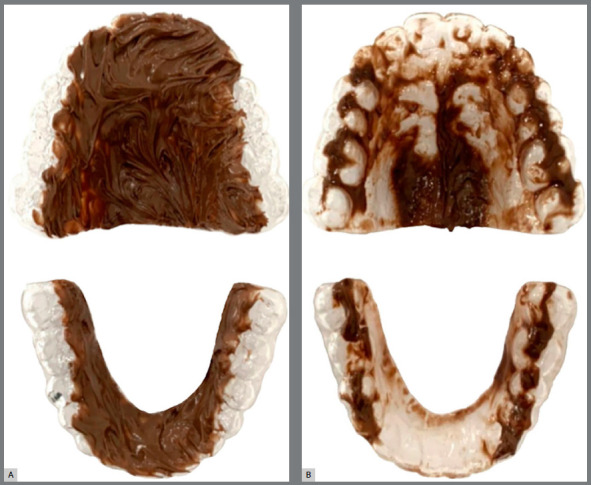
Retainers coated with molten chocolate: A) Before and; B) After evaluation of the tongue position during swallowing. The missing part of the chocolate indicated the tongue contact area.

Evaluation of the tongue position during swallowing was performed using indirect retainers coated with molten chocolate before and after the tongue therapy.^22^ ^,^ ^23^ Maxillary and mandibular clear retainers, which were covered with a thin layer of molten chocolate, were placed in the maxillary and mandibular arches. Then the participants were asked to return their tongue to a resting position and keep their mouth naturally relaxed for swallowing.^24^ Tongue-palate contact was revealed. The missing chocolate part was used to indicate the tongue contact (Fig 1).

### CUSTOMIZED BONDED SHARK TOOTH-LIKE SPURS (JAWS) PROCEDURE

**Figure 2:**
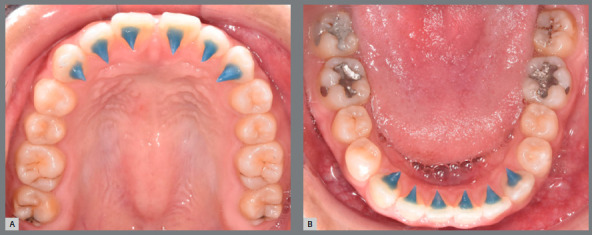
JAWs were placed on the maxillary (A) and mandibular (B) anterior teeth.

All participants received tongue reeducation with JAWs for three months before orthodontic treatment. The JAWs were placed on the lingual surfaces of the maxillary and mandibular anterior teeth. Compomer cement (Ultra Band-Lok™ push syringe in blue shade, Reliance Orthodontic Products, Inc., Itasca, Illinois, USA) was used to fabricate JAWs (3-mm long) with sharp tips (Fig 2). No other therapeutic appliances nor myofunctional therapy were used during the observation period. 

### CEPHALOMETRIC MEASUREMENTS

**Figure 3:**
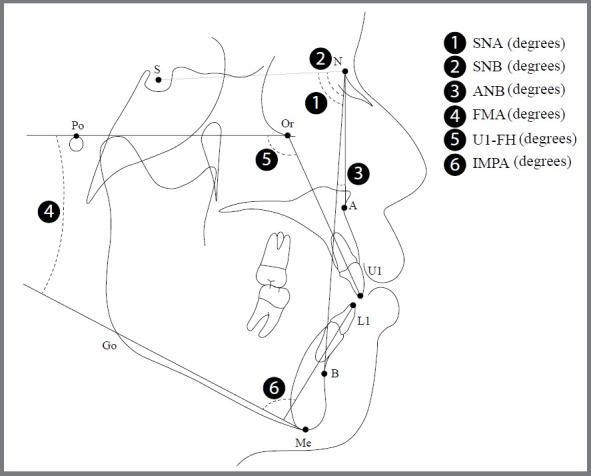
Cephalometric measurements used in the study.

Lateral cephalometric analyses were conducted at baseline (T0) and three months after tongue therapy with the JAWs (T3). The cephalometric analysis of the dentoalveolar measurements is shown in Figure 3. The Dolphin Imaging Program (v. 11.8 Imaging Program, Chatsworth, California, USA) was used for data collection and generation. 

### 3D DIGITAL DENTAL MODEL MEASUREMENTS

**Figure 4:**
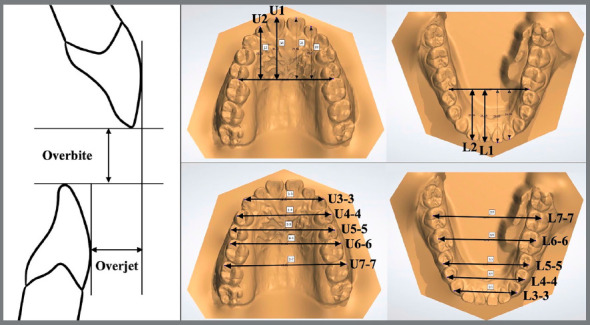
Reference points on 3D digital dental model landmarks used in the study.

3D digital dental models were obtained from a 3D scanner (3Shape R700, 3Shape A/S, Copenhagen, Denmark) at baseline (T0); one (T1), two (T2), and three months (T3) after JAWs use. The following measurements were performed; overbite, overjet, and arch length discrepancy. In the maxilla (U) and mandible (L), the intercanine (3-3), first (4-4), and second (5-5) interpremolar and, first (6-6) and second (7-7) intermolar widths were measured. In the anteroposterior dimension, the distance between the incisors to a constructed line mesial to the first molars was also assessed using OrthoAnalyzer^TM^ 3D software, 2013 version (3Shape A/S) for the 3D digital dental model analysis (Fig 4). 

### MEASUREMENT RELIABILITY

To test the measurement reliability, the same investigator repeated the measurement of ten patients after three weeks. The intraclass correlation coefficient (ICC) for the angular measurements ranged from 0.92 to 0.99, and that for the linear measurements ranged from 0.93 to 0.99 (95% confidence interval). The values obtained from the angular and linear measurements in the first and second measurements were tested using the *t*-test, to study the systematic error. None of the 26 variables analyzed presented a statistically significant systematic error.

### STATISTICAL ANALYSIS

Statistical analysis was conducted using SPSS v. 23.0.0 software (SPSS Inc. Chicago, IL, USA). Data normality was assessed using the Shapiro-Wilk test. The data represented normal distributions. Therefore, parametric statistical analysis was used in the present study. Paired *t*-test and repeated measures ANOVA test were used to compare changes over time within groups, and Pearson correlations were used to relate the influential factors related to the amount of overbite change. The significance level was set at *p*<0.05.

## RESULTS

For the demographic data, the study observed 12 (52.2%) Class I, 8 (34.8%) Class II, and 3 (13%) Class III malocclusions. Moreover, 14 (60.9%) hyperdivergent and 9 (39.1%) normal vertical skeletal patterns were found.

**Figure 5:**
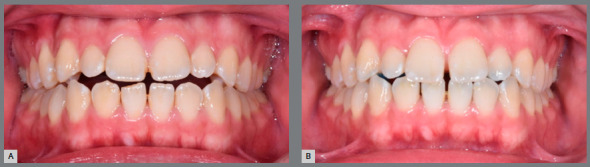
Dentoalveolar changes after using tongue therapy with JAWs: Pretreatment (A) and post-treatment (B).

**Figure 6:**
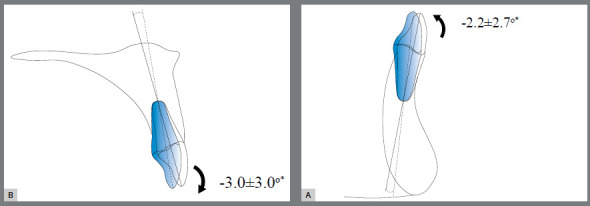
Diagram of the dentoalveolar changes following tongue therapy with JAWs on the maxilla (A) and on the mandible (B).

Significant dentoalveolar changes were observed (Fig 5). Most of the significant dentoalveolar changes occurred within the first month (T1) following the use of JAWs (Tables 1, 2). In the vertical dimension, a significant increase in the overbite (1.0±0.6 mm) was observed. The minimum difference detected in the intragroup comparison was 0.3 mm, while the maximum difference was 2.3 mm. The significant changes in the overbite were observed during the initial follow-up (T0-T1) of one month. In the anteroposterior dimension, a decrease in overjet (0.2±0.3 mm) was found. Significant retroclinations of the maxillary (3.0±3.0°) and mandibular (2.2±2.7°) incisors were observed. A diagram of the main dentoalveolar changes is shown in Figure 6.

**Table 1:** Comparison of cephalometric variables at baseline (T0) and three months (T3) after tongue reeducation with JAWs.

Variables	T0	T3	(T3-T0)	t	p
	Mean (SD)	Mean (SD)	Mean (SD)		
Skeletal variables					
SNA (degrees)	83.6 (2.9)	83.5 (3.0)	0.1 (1.4)	0.474	0.643
SNB (degrees)	80.6 (3.6)	80.5 (3.6)	0.1 (0.9)	0.057	0.995
ANB (degrees)	3.0 (2.8)	3.0 (2.8)	0.1 (0.3)	0.364	0.722
FMA (degrees)	30.3 (6.7)	30.0 (6.6)	0.3 (0.6)	1.595	0.133
Dental variables					
U1-FH (degrees)	120.1 (6.2)	117.2 (6.4)	-3.0 (3.0)	3.895	0.002**
IMPA (degrees)	93.6 (8.6)	91.3 (9.3)	-2.2 (2.7)	3.184	0.007**

Values are presented as mean ± standard deviation (SD) or p-value.

Paired t-test, t: p-value significant at *p<0.05; **p<0.01; ***p<0.001.

**Table 2:** Comparison of 3D digital dental model variables at different time points: baseline (T0), one month (T1), two months (T2), and three months (T3) after tongue reeducation with JAWs.

Variables	T0	T1	T2	T3	(T3-T0)	F	p			
	Mean (SD)	Mean (SD)	Mean (SD)	Mean (SD)	Mean (SD)		T0-T1	T1-T2	T2-T3	T0-T3
Linear										
Vertical										
OB (mm)	-1.4 (1.4)	-0.4 (1.6)	-0.4 (1.6)	-0.4 (1.6)	1.0 (0.6)	54.940	0.000***	0,146	1.000	0.000***
Anteroposterior										
OJ (mm)	2.2 (2.1)	2.5 (2.1)	2.5 (2.1)	2.5 (2.1)	0.2 (0.3)	18,729	0.000***	1.000	1.000	0.000***
U1 (mm)	27.6 (2.2)	27.0 (2.1)	27.0 (2.1)	27.0 (2.1)	-0.6 (0.3)	86,542	0.000***	0.680	0,266	0.000***
U2 (mm)	23.7 (2.4)	23.3 (2.3)	23.3 (2.3)	23.3 (2.3)	-0.4 (0.2)	81,513	0.000***	0,747	0,103	0.000***
L1 (mm)	23.2 (2.2)	22.3 (2.1)	22.3 (2.1)	22.4 (2.2)	-0.8 (0.1)	454,603	0.000***	1.000	0,184	0.000***
L2 (mm)	21.6 (2.4)	21.1 (2.4)	21.0 (2.4)	21.1 (2.4)	-0.6 (0.3)	52,012	0.000***	0,503	0,451	0.000***
Transverse										
U3-3 (mm)	36.1 (2.1)	35.7 (2.0)	35.7 (2.0)	35.7 (2.0)	-0.4 (0.4)	28,327	0.000***	0,313	0,607	0.000***
U4-4 (mm)	44.1 (2.6)	43.9 (2.5)	43.9 (2.5)	43.9 (2.6)	-0.2 (0.3)	4,242	0.020*	0,727	0,361	0.031*
U5-5 (mm)	48.8 (3.8)	49.0 (3.7)	49.0 (3.7)	49.0 (3.7)	0.2 (0.3)	6,738	0.016*	0,712	0,723	0.013*
U6-6 (mm)	54.8 (2.2)	55.0 (2.2)	55.0 (2.2)	55.0 (2.2)	0.1 (0.2)	7,878	0.003**	0,058	0,201	0.011*
U7-7 (mm)	60.2 (2.2)	60.2 (2.3)	60.2 (2.3)	60.2 (2.2)	0.0 (0.2)	1,324	1.000	1.000	1.000	1.000
L3-3 (mm)	28.3 (3.3)	28.0 (3.3)	28.1 (3.2)	28.1 (3.2)	-0.3 (0.3)	14,023	0.000***	0.099	0.336	0.000***
L4-4 (mm)	36.7 (3.2)	36.5 (3.2)	36.5 (3.2)	36.6 (3.1)	-0.1 (0.3)	3,538	0,341	1.000	0.754	0,765
L5-5 (mm)	40.8 (3.1)	40.9 (3.3)	40.9 (3.3)	42.0 (3.2)	0.2 (0.5)	2,292	0,718	1.000	1.000	0.160
L6-6 (mm)	46.7 (3.4)	46.6(3.4)	46.6 (3.4)	46.6 (3.4)	0.3 (0.5)	2,203	0,881	0,281	0,833	0.857
L7-7 (mm)	52.5 (2.5)	52.6 (2.5)	52.6 (2.5)	52.6 (2.5)	0.1 (0.4)	2,202	0,501	1.000	1.000	0,633

Values are presented as mean ± standard deviation (SD) or p-value.

Repeated measure ANOVA test, F: p-value significant at *p<0.05; **p<0.01; ***p<0.001.

**Figure 7:**
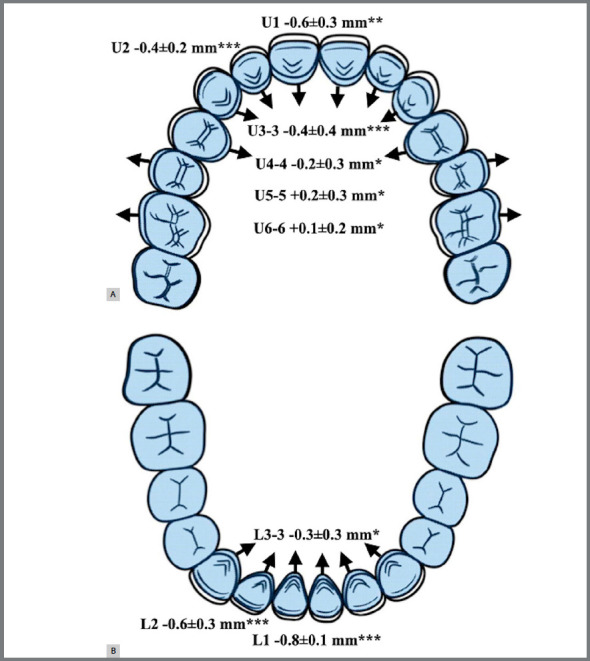
Diagram of the dentoalveolar changes following tongue therapy with JAWs on the maxilla (A) and on the mandible (B).

In the transverse dimension, a decrease in the maxillary (0.4±0.4 mm) and mandibular (0.3±0.3 mm) intercanine width as well as in the maxillary first premolar width (0.2±0.3 mm) was observed. However, an increase in the maxillary second premolar (0.2±0.3 mm) and first molar width (0.1±0.2 mm) was observed. A significant retroclination of the maxillary (0.6±0.3 mm) and mandibular (0.8±0.1 mm) anterior incisors was also observed. A diagram of the main dentoalveolar changes is shown in Figure 7. 

Moreover, a significant correlation between the arch length discrepancy and the overbite changes was observed (r= 0.456, *p*<0.05) (Table 3). 

**Table 3:** Correlation between treatment variables and overbite change.

Variables	Overbite change	
	r	p
Arch length discrepancy	0.453	0.043*
Overbite	0.068	0.759
FMA (degrees)	0.041	0.883
ANB (degrees)	0.274	0.323

Pearson’s test, r: p-value significant at *p<0.05.

During the three-month observation period, 36 of a total of 276 (4.4%) JAWs were fractured or dislodged. Breakage of the spurs was mainly concentrated in the T1 (75%), followed by T2 (22.2 %) and T3 (2.8%), respectively. Moreover, the rate of breakage was higher in the maxilla (66.67%) than in the mandible (33.33%).

No significant skeletal changes were observed throughout the observation period.

## DISCUSSION

Several previous studies have reported dentoalveolar changes resulting from various AOB treatment modalities in growing patients, due to the effect of the treatment combined with the growth potential.^3^ ^,^ ^8^ ^,^ ^12^ ^,^ ^18^ ^,^ ^19^ ^,^ ^25^ ^,^ ^26^ Furthermore, numerous literature reviews have emphasized that the correction of a functional habit during AOB treatment leads to increased long-term stability.^8^ ^,^ ^9^ ^,^ ^13^ However, according to our knowledge, only a few case reports on the usage of tongue spurs in adult patients are available,^13^ and none of them provided information about the dentoalveolar effects. Therefore, the dentoalveolar changes after tongue therapy in adult AOB patients are unclear.

In the present study, the analysis of dentoalveolar changes followed by the correction of tongue habits with JAWs in adult patients was performed. Significant improvement in the inclination of both maxillary and mandibular anterior teeth was observed, which might have resulted in significant changes in the overjet and overbite. These results are in agreement with previous studies that investigated the effectiveness of tongue reeducation appliances in growing patients during early treatment.^3^ ^,^ ^19^ ^,^ ^20^ These results suggested that the use of JAWs has the potential to change the tongue-thrusting habit in adult patients, therefore changing the effects of tongue position on dentition. The mechanism of the spurs therapy in tongue-thrusting has been hypothesized by Meyer-Marcotty et al.,^17^ according to which neural pathways allow a change in the anterior tongue rest posture through the lingual nerve, (afferent, or sensory), and the hypoglossal nerve (efferent, or motor).^13^ Hence, it is inferred that spurs alter orofacial function, theoretically resulting in a change in the dentoalveolar form.^13^ Therefore, the use of JAWs might potentially correct the tongue posture and function by stimulating proprioceptive receptors on the pain pathway, then generating the learning process in the brain. Neuromuscular adaptation occurs, then sending the feedback to result in a new normal tongue rest posture, leading to a secondary effect on the dentoalveolar form that allows the spontaneous lingual movement of incisors and canines, thus improving the AOB.^17^ The results of the present study suggested that the use of JAWs in adult patients generates similar learning mechanisms in the brain as the ones observed in growing patients, thus allowing for spontaneous dentoalveolar changes.

The 3D model analysis showed particular patterns of dentoalveolar change observed for the anterior and posterior regions, followed by the use of JAWs. In the anterior region, it was observed a significant amount of lingual movement of the anterior teeth, in both maxillary and mandible arches, thus resulting in a decrease of dental arch length. Theoretically, this change might be caused by a more posterior position of the tongue following the use of JAWs. These dentoalveolar changes have been reported in previous studies.^3^ ^,^ ^17^ In the posterior region, a significant increase in both maxillary second premolar and first molar widths was observed, representing the expansion of the maxillary dental arch. However, no changes in the dental arch width in the posterior region of the mandible were noted. These changes might be caused by a more posterosuperior tongue position following the use of JAWs. These dentoalveolar changes are considered a compensatory response to the related new adaptation tongue position and function changes that took place after the use of JAWs. It is also inferred that this new adaptive tongue position results in a secondary effect in the dentition, with a decrease of the arch width in the anterior region and an increase in both maxillary second premolar and first molar width. The results are in agreement with previous studies that used bonded spurs for the correction of tongue habit in growing patients, and observed a posterior resting tongue position.^20^ Taslan et al.^27^ reported that the arch changes might have been due to an alteration in the resting tongue position and a resulting change in tongue pressure on the teeth after tongue therapy with crib appliances, reporting significant increase in resting tongue pressure on the maxillary first molar and decrease on the mandibular incisors.

An important finding of the present study was that the most significant dentoalveolar changes occurred in the initial month of therapy with JAWs. This result suggests that an immediately nociceptive reflex is established to a new tongue rest posture and function mode after JAWs insertion, resulting in a physiologic dental drift and dental form changes.^13^ Moreover, since no significant dentoalveolar changes were observed during the follow-up periods, we might assume that the nociceptive reflex created by the presence of JAWs can be maintained. Therefore, it might be assumed that the use of JAWs might play an important role in the stability of changes following the treatment of AOB cases. Although various retention periods have been suggested following the use of spurs, little is known about the adequate retention periods for achieving long-term stability.^13^ ^,^ ^17^ According to Justus,^13^ spurs should remain in place for at least one year, to ensure that the parafunctional tongue-thrusting habit has been corrected. Haryett et al.^28^ also recommended that the spurs should remain for at least six months after the positive anterior overbite is achieved. Moreover, Meyer-Marcotty et al.^17^ reported that bite deepening occurred within the initial six months and suggested that the spurs were then left in place for only three months to stabilize the tongue rest position. However, it is unknown if the changes in the position and function of the tongue can be maintained after the removal of the JAWs. Therefore, further studies are necessary to evaluate the effects of JAWs on the long-term stability after AOB cases treatment.

In the present study, the tongue position during swallowing was assessed indirectly using clear retainers coated with molten chocolate. Although this approach was relatively simple and easy to be performed compared to the conventional palatography using electronic systems, it lacks validation of the accuracy and reliability of the method. This consists in a limitation of the study to correctly identify the tongue posture and function. However, further studies are necessary to assess the accuracy and reproducibility of this method. 

Another limitation of the study was the absence of a control group to assess the overall dentoalveolar changes following the use of JAWs. Since only adult patients with non-growth potential were included, the dentoskeletal changes following the use of JAWs were compared with the patients’ initial baseline values. 

In the present study, a positive correlation between the arch length discrepancy and the dentoalveolar changes was observed after the use of JAWs. This result reinforces the potential importance of JAWs for the correction of the tongue position and function in AOB cases. Since the arch length discrepancy in this study was defined as the initial spacing, it is possible to infer that the more severe the AOB, the more benefit from the tongue therapy with JAWs can be achieved. Therefore, the use of JAWs might be considered as an adjunctive treatment modality for the correction of severe AOB cases, such as those requiring surgical correction, to improve the long-term stability of changes. However, the effects of JAWs on the long-term stability of changes following orthodontic treatment should be further investigated. 

No correlation was observed between dentoalveolar changes and the initial vertical and anteroposterior skeletal pattern. However, the size of JAWs for the present study was standardized to 3-mm long for all subjects; therefore, it might have limited their effectiveness in different vertical and anteroposterior skeletal patterns. To date, we found no studies assessing the effects of spur size on the skeletal patterns of AOB. Moreover, most of the commercially available bonding spurs present with the same shape and size to fit all types of problems.^3^ ^,^ ^12^ Although the negative result observed, the need to adjust the shape and size of JAWs following the skeletal patterns to obtain an efficient tongue habit correction is suggested. However, further studies are necessary to investigate the effect of JAWs sizes among different skeletal patterns.

## CONCLUSIONS

A spontaneous 3D dentoalveolar change occurred following the first-month therapy with JAWs. The retroclination of the anterior, combined with the expansion of posterior teeth suggests a posterosuperior change in the tongue position. These changes suggest alterations in the tongue position. However, further studies are necessary to investigate the effect of JAWs on the tongue position and function.
